# Prevalence of sexual, physical and emotional abuse in the Norwegian mother and child cohort study

**DOI:** 10.1186/1471-2458-13-186

**Published:** 2013-03-02

**Authors:** Marie Flem Sørbø, Hilde Grimstad, Johan Håkon Bjørngaard, Berit Schei, Mirjam Lukasse

**Affiliations:** 1 Department of Public Health and General Practice, Faculty of Medicine, Norwegian University of Science and Technology, (NTNU), Postbox 8905, Trondheim, N-7491, Norway; 2 St. Olav’s Hospital Trondheim, Forensic Department and Research Centre, Brøset, Norway; 3 Department of Gynecology, St. Olavs University Hospital, Trondheim, Postbox 3250, Trondheim, Sluppen N-7006, Norway; 4 Department of Health, Nutrition and Management, Faculty of Health Sciences, Oslo and Akershus University College of Applied Sciences, Postboks 4 Alnabru, St. Olavs plass, Oslo, N-0130, Norway

**Keywords:** Emotional abuse, Sexual abuse, Physical abuse, Prevalence, The Norwegian mother and child cohort study

## Abstract

**Background:**

Abuse of women occurs in every society of the world. Increased information about the prevalence in industrialized countries, like Norway, is required to make strategies to prevent abuse. Our aim was to investigate the prevalence of self-reported sexual, physical and emotional abuse in a large obstetric population in Norway, and the associations between exposure to adult abuse, socio-demographics and other characteristics.

**Methods:**

Our study is based on the Norwegian Mother and Child (MoBa) Cohort study, conducted by the Norwegian Institute of Public Health. The current study included 65,393 women who responded to two extensive postal questionnaires during pregnancy. Any adult abuse is defined as being exposed to one or more types of adult abuse, any child abuse is defined as being exposed to one or more types of child abuse, and any lifetime abuse is defined as being exposed to abuse either as a child and/or as an adult. Perpetrators were categorized as known or stranger.

**Results:**

Overall, 32% of the women reported any lifetime abuse, 20% reported any adult abuse, 19% reported any child abuse and 6% reported abuse both as adults and as children. Emotional abuse was the most frequently reported type of abuse both as adults (16%) and children (14%). Adult sexual abuse was reported by 5% and child sexual abuse by 7%. Physical abuse was reported by 6% as adults and by 6% as children. Approximately 30% of those reporting adult or child abuse reported exposure to two or three types of abuse. Five percent of the women reported exposure to any abuse during the last 12 months. For all types of abuse, a known perpetrator was more commonly reported. Logistic regression showed that being exposed to child abuse, smoking and drinking alcohol in the first trimester of pregnancy, living alone, and belonging to the eldest age group were significantly associated with being exposed to any adult abuse.

**Conclusion:**

The reported prevalence of any lifetime abuse was substantial in our low-risk pregnant population. Antenatal care is an opportunity for clinicians to ask about experiences of abuse and identify those at risk.

## Background

Every fifth woman in the world faces some type of abuse during her lifetime, in some cases leading to serious injury or death [1]. Abuse of women and girls is widely recognized as a major public health problem and as a violation of women's rights. The United Nations (UN) defines violence against women as *'any act of gender-based violence that results in, or is likely to result in, physical, sexual or mental harm or suffering to women, including threats of such acts, coercion or arbitrary deprivation of liberty, whether occurring in public or in private life*’ [2].

There is an increasing awareness of the extent of emotional, physical and sexual abuse against women, particularly during childbearing periods, and of their possible negative consequences. Prevalence studies of abuse and identification of risk factors provide valuable information for the prevention of violence against women. The prevalence of reported abuse varies considerably, depending among other things on definitions used, study design, the population studied, and the response rate achieved [3-8]. Standardization of research has been requested to facilitate comparisons among studies on abuse [4,7]. The World Health Organization (WHO) carried out a multi-country study on domestic violence between 2000 and 2003 where one aim was to collect internationally comparable data by using standardized survey methods [9]. Between 15% and 71% of women from the ages of 15 to 49 years reported lifetime sexual and/or physical partner violence, and 4% to 54% of respondents experienced this violence within one year prior to the study [9]. Findings from the WHO study showed that the prevalence of abuse was much lower in industrialized environments than in any other study settings, possibly suggesting that variations of prevalence can be related to cultural and economic differences in the patterns of abuse. Prevalence of pregnancy-related abuse also varies. In a review article from the United States, prevalence of abuse during pregnancy was reported to range from 0.9% to 20.1% [7]. A lower prevalence is expected when information is collected from self-administered questionnaires compared with personal interviews and a higher prevalence with well qualified interviewers, use of structured screen and with repeated questioning [7,10,11]. Studies from industrialized countries, including Norway, also reveal high levels of abuse, but the prevalences reported in the various studies are difficult to compare due to methodological differences, the studies are usually small, performed in special age groups, or differing in types of abuse are investigated. The first national study in Norway of partner violence on women from the ages of 20 to 55 years showed that 27% had experienced abuse by their partner and 6% in the year before the study [12]. In another Norwegian study among approximately 7000 senior students (about 18 years of age) in secondary school, sexual abuse was reported by 22% of the women [13]. More studies of abuse of Norwegian women are required to devise prevention strategies, and a population-based approach will give more and better information to the field. To our knowledge, the current study is the largest population-based study of emotional, sexual and physical abuse reported by pregnant women in Norway. Our primary aim was to investigate the prevalence of sexual, physical and emotional abuse reported by a large pregnant population in Norway. The secondary aim was to investigate the identity of the perpetrator, and to compare women reporting adult abuse with those who did not with regard to socio-demographics and other characteristics.

## Methods

### Study population

**Figure 1:**
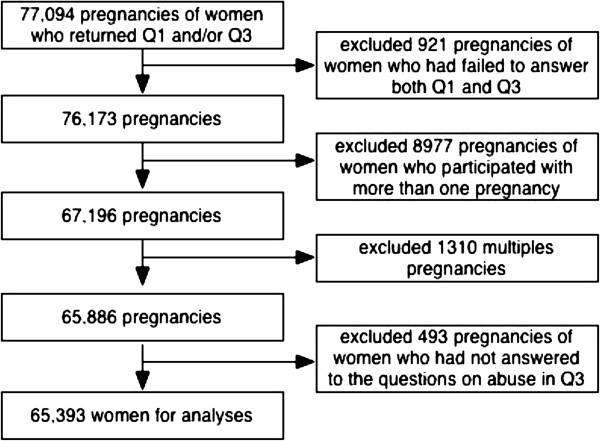
Flow-chart of inclusion in the study population.

**Figure 2:**
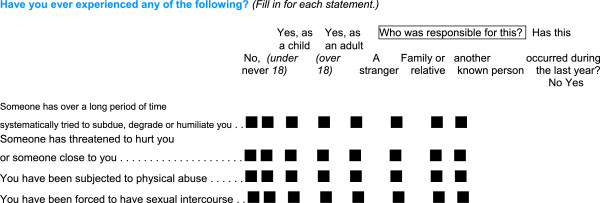
Questions on abuse and perpetrators in questionnaire 3 in the Norwegian mother and child cohort study, 1999–2009.

Our study is based on the Norwegian Mother and Child Cohort Study (MoBa), which is a prospective population-based pregnancy cohort study conducted by the Norwegian Institute of Public Health [14]. The inclusion period was from 1999 to 2008, and 90,700 mothers and 108,000 children participated in the MoBa study. Hospitals with more than 100 births annually were invited to collaborate and 70% of all pregnant women in Norway during this period were invited to participate. The overall response rate was 38.5%. All pregnant women in Norway are offered a routine ultrasound screening at week 18 of gestation at their local hospital [14]. Together with the ultrasound appointment, the women received a postal invitation that included an informed consent form, the first questionnaire and an information brochure. A detailed protocol of the study including the consent can be found elsewhere (http://www.fhi.no/morogbarn). Women who agreed to participate received three extensive self-administrated questionnaires by post during pregnancy. The MoBa sample has been described in more detail elsewhere [14,15]. Data from the questionnaires are linked to the Medical Birth Registry of Norway, which has kept records of all deliveries in Norway since 1967. This register is based on a standardized form completed by midwives shortly after delivery. Pregnancy was the unit of observation in the MoBa survey; while in the current study the unit of observation was the woman. Figure 1 shows a flow-chart of those excluded from the current study. We merged Questionnaires One and Three, and only women who had filled in both questionnaires were included. For women who participated with more than one pregnancy, only information from their first pregnancy was included. Only singleton pregnancies were included, and only women who had answered a minimum of one of the abuse questions (Figure 2) in Questionnaire Three were included, leaving a total of 65,393 women for the analyses. The current study is based on version 4 of the data files released for research in 2008. Written informed consent was obtained from each participant at recruitment. The study was approved by The Regional Committee for Medical Research Ethics in South-Eastern Norway.

### Abuse variables

The third MoBa questionnaire was posted around Week 30 of gestation. It included four questions on abuse. Figure 2 shows the questions on abuse, and the response options provided. The two questions on emotional abuse are similar to two of the three questions on emotional abuse in the validated NorVold questionnaire [16], which is used in a review article and in other studies in the Nordic countries [17-20]. We merged the questions of emotional abuse into one variable in the analyses. The question on sexual abuse with response options in our study, is a modified version of the sexual abuse question in the Abuse Assessment Screen (ASS) [21], which is an abuse screening tool, and has been used in other Scandinavian prevalence studies and in an English study of pregnant populations [6,10,11]. The question of physical abuse is not validated. Women could respond “no never” to the various types of abuse, or “yes” as an adult (≥ 18 years) and/or as a child (< 18 years) to the various types of abuse. Women who answered yes to at least one of the adult abuse questions were defined as having suffered from any adult abuse. Likewise, women who responded yes to one or more of the child abuse questions were defined as having suffered from any child abuse. Those who responded yes to any abuse either as an adult or as a child were defined as suffering from any lifetime abuse. Women could also indicate whether they experienced abuse during the last 12 months.

### Perpetrators

In Questionnaire Three, women were given the opportunity to reveal who committed the abuse: a stranger, family/relative, or known other (Figure 2). The two latter categories were merged and hence we used the two categories of stranger and known in our analysis. Through the way in which questions about abuse and perpetrators were expressed, women could indicate abuse both as an adult and as a child, and by one, two or no perpetrators. To be able to relate perpetrators to adult or child abuse, respectively, we used a segregated category of *only* adult abuse and *only* child abuse for the different types of abuse. To achieve this, we subtracted those who had responded yes to both child and adult abuse from the adult abuse category and likewise for the child abuse category. In addition, we have one category for women reporting exposure to both adult and child abuse. Table 1 shows the numbers of women reporting abuse only as a child, only as an adult, or both. These are also the categories needed to relate information about exposure to adult or child abuse during the last 12 months.

**Table 1:** Types of abuse and groups of exposure, Norwegian mother and child cohort study, 1999-2009

	**Emotional abuse**		**Physical abuse**		**Sexual abuse**		**Any abuse**	
	**N**	**(%)**	**N**	**(%)**	**N**	**(%)**	**N**	**(%)**
**Child only**	6601	(10.1)	3151	(4.8)	4072	(6.2)	8143	(12.5)
**Adult only**	8272	(12.6)	3276	(5.0)	3039	(4.6)	10891	(16.7)
**Child and adult**	2467	(3.8)	459	(0.7)	473	(0.7)	4121	(6.3)
**No abuse**	48053	(73.5)	58507	(89.5)	57809	(88.4)	44253	(67.7)

### Other variables

Background information such as age, civil status, education, parity, body mass index (BMI), and use of tobacco and alcohol during the first trimester were collected from Questionnaire One (Table 2). Information about education was categorised into four groups: primary school (9 years), secondary school (12 years), higher education (college or university) up to 4 years, and higher education more than 4 years. Information about parity was based on number of self-reported previous deliveries >21 weeks of gestation, and categorized into women never giving birth (P0), and women giving birth previous to this pregnancy (P+). Civil status was redefined into three groups: married, not married but cohabitee, and living alone. BMI was calculated from self-reported information about height and weight pre-pregnancy. Age was divided into five groups. We wanted to compare with the largest age group – hence we chose the age group 30–34 as reference. Smoking was recoded into three categories: no smoking, sometimes, and daily in the first trimester. Alcohol use first trimester was re-categorized into: never, less than once a week (one alcohol unit), and 2–7 days a week. All background information was reported at Week 18 of gestation.

**Table 2:** Socio-demographics and risk factors in relation to different types of adult abuse (N = 65,393)

		**No abuse**		**Emotional abuse**		**Physical abuse**		**Sexual abuse**		**Any abuse**	
		**n = 52,396**	**80%**	**n = 10,739**	**16%**	**n = 3,735**	**6%**	**n = 3,512**	**5%**	**n = 12,997**	**20%**
		**n**	**%**	**n**	**%**	**n**	**%**	**n**	**%**	**n**	**%**
**Age (yr)**	n*										
14-19	947	800	85	124	13	35	4	33	4	147	16
20-24	8049	6551	81	1214	15	425	5	431	5	1498	19
25-29	23614	19434	82	3444	15	1158	5	1096	5	4180	18
30-34	26354	20954	80	4449	16	1593	6	1475	6	5400	21
>35	6428	4657	72	1507	23	523	8	476	7	1771	28
missing	1	0	0	1	0	1	0	1	0	1	0
**Civil status**											
married	31642	2617	83	4451	14	1359	4	1462	5	5464	17
cohabiting	31320	24653	79	5531	18	2068	7	1785	6	6667	21
not cohabiting	2072	1297	63	673	33	285	14	240	7	775	37
missing	359	268	<1	84	<1	23	<1	25	<1	91	<1
**Education**											
primary	1638	1176	72	404	25	171	10	134	8	462	28
secondary	19297	14886	77	3749	19	1386	7	1206	6	4411	23
≤ 4 yr uni	37739	31298	83	5150	14	1630	4	1697	5	6441	17
> 4 yr uni	4340	3198	74	996	23	370	9	310	7	1142	26
missing	2379	1838	4	440	4	178	5	165	5	541	4
**Parity**											
P 0	33913	27494	81	5261	16	1748	5	1687	5	6419	19
P +1	31480	24902	79	5478	17	1987	6	1825	6	6578	21
missing	0										
**BMI**											
<20	7947	6389	80	1274	16	481	6	490	6	1558	20
20-24,9	35576	28780	81	5622	16	1945	6	1758	5	6816	19
25-29,9	13934	11102	80	2350	17	795	6	753	5	2832	20
>30	6074	4690	77	1156	19	392	7	397	7	1384	23
missing	1862	1455	3	337	3	122	3	114	3	407	3
**Smoking 1st trim.**											
no	58934	47953	81	8983	15	2974	5	2940	5	10981	19
sometimes	2010	1460	73	471	23	191	10	127	6	550	27
daily	3956	2600	66	1194	30	532	13	411	10	1356	34
missing	493	383	<1	91	<1	38	1	34	1	110	<1
**Alcohol 1st trim.**											
Never	48498	39151	81	7767	16	2666	6	2503	5	9347	19
<1 /week	8377	6425	77	1529	18	586	7	596	7	1952	23
2-7 /week	52	30	58	18	35	5	10	8	15	22	42
missing	8466	6790	13	1425	13	478	13	405	12	1676	13
**Child abuse**											
no	53129	44253	83	7366	14	2507	5	2287	4	8876	17
yes	12264	8143	66	3373	28	1228	10	1225	10	8877	17
missing	0	0	0	0	13	0	13	0	0	0	0

* Total number of women in each category.

### Data analysis

Descriptive statistics were presented for all women. Logistic regression analyses were performed on any adult abuse as crude (unadjusted) and adjusted odds ratios (ORs). In the adjusted model, the various categories of socio-demographic characteristics (age, education, civil status, and parity) and other characteristics (BMI, smoking and alcohol consumption, child abuse) were included. The results from the logistic models were presented with 95% confidence intervals (95% CI) and analysed for complete cases only. The data programme PASW statistical 18 was used in the analyses.

## Results

### Prevalence of different types of abuse

**Figure 3:**
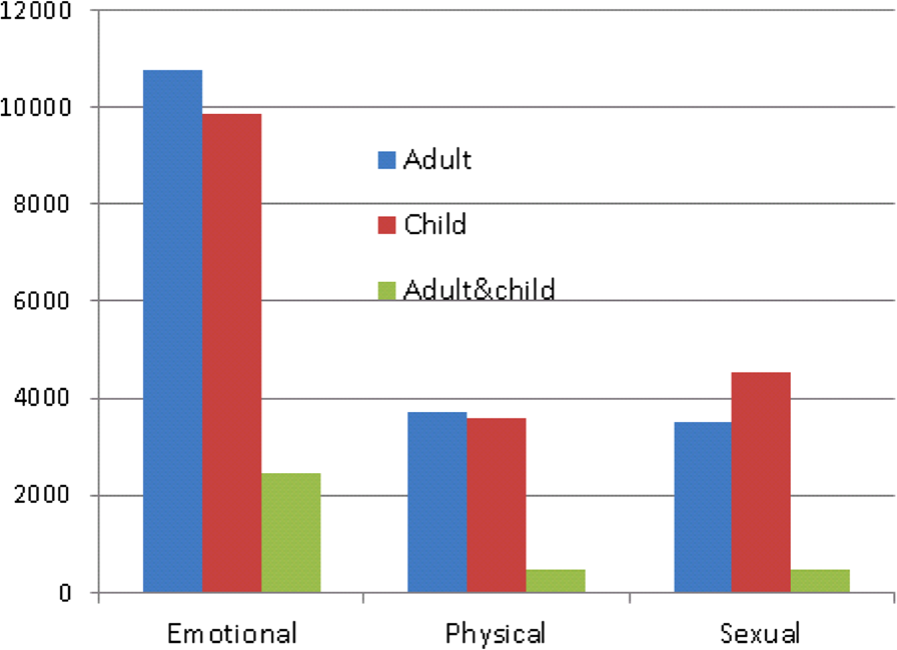
Number of women reporting different types of abuse at various age groups.

**Figure 4:**
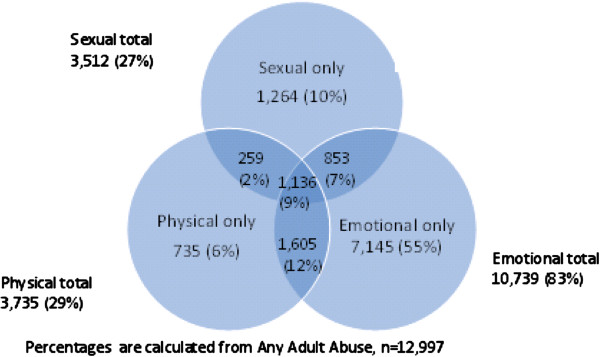
Types of adult abuse (n = 12,997) and overlapping categories.

Overall, any lifetime abuse (which includes adult and child abuse except those exposed to both adult and child abuse) was reported by 32% of the women, 20% reported any adult abuse, 19% any child abuse, and 6% reported both any adult abuse and any child abuse. Figure 3 shows reported types of abuse according to the different age groups. Among those reporting any adult abuse, sexual and physical abuse were reported by 27% (3512) and 28% (3735), respectively, and emotional abuse by 83% (10,739). Among women reporting any child abuse, 37% (4545) reported sexual abuse, while 29% (3610) reported physical abuse and 74% (9865) reported emotional abuse. Of those reporting any adult abuse, 30% had been exposed to two or more types of abuse (Figure 4). The same occurred among women reporting any child abuse, where 31% reported two or more types of abuse. Among women exposed to emotional abuse as an adult, 23% had also experienced emotional abuse as a child; the absolute numbers are shown in Figure 3. Of the women reporting sexual abuse as an adult, 14% also reported child sexual abuse, whereas 12% who suffered physical abuse as an adult also reported child physical abuse. Of those who experienced any adult abuse, 32% reported any child abuse.

Five percent of the study population indicated having experienced any abuse in the last 12 months. Among those reporting physical abuse only as an adult, 8% reported physical abuse in the last 12 months. Furthermore, 3% of those reporting sexual abuse only as an adult also reported sexual abuse in the last 12 months, while among women reporting emotional abuse only as an adult, 22% reported emotional abuse in the last 12 months. The questions on abuse during the last 12 months had responses missing for between 73% to 88% of the different types of abuse.

### Perpetrators

Thirty two percent of the women in the study reported any abuse: of whom nearly all (98%) also reported who committed the abuse. Overall, 29% reported a known perpetrator, 5% reported a stranger, and 3% reported being exposed to abuse from both a known perpetrator and a stranger.

### Associations between background information and exposure to adult abuse

**Figure 5:**
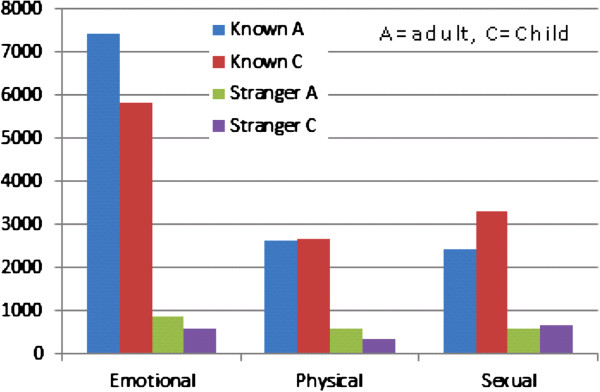
Number of reported perpetrators according to child and adult abuse.

Figure 5 shows the reported perpetrators of the different types of abuse according to only adult abuse and only child abuse. Table 3 shows the crude and adjusted logistic regression analysis on any adult abuse according to background information. Some factors were strongly associated with being exposed to adult abuse while other factors showed less or no association. In the adjusted analyses, BMI and parity were not substantially associated with any adult abuse. Smoking daily and drinking alcohol weekly in the first trimester of pregnancy or being exposed to child abuse were associated with being exposed to adult abuse (OR =1.6, [95% CI: 1.5, 1.8]; OR 2.5, [95% CI: 1.4, 4.6]; and OR =2.4, [95% CI: 2.3, 2.5], respectively). Married or cohabiting women were less likely to report adult abuse compared with women living alone. Women at 35 years or older were more likely to have been exposed to adult abuse than the younger women. The unadjusted logistic regression was in the main confirmed by the adjusted results.

**Table 3:** Logistic regression analysis on any adult abuse according to socio-demographics and risk factors

	*n	**Crude OR**	**95% CI**	**Adjusted OR**	**95% CI**
**Age**					
14-19	111	0.8	(0.7-1.0)	0.3	(0.2-0.4)
20-24	1120	0.9	(0.8-0.9)	0.6	(0.5-0.6)
25-29	3317	0.8	(0.8-0.9)	0.8	(0.7-0.8)
30-34**	4415	1.0		1.0	
≥ 35	1437	1.5	(1.4-1.6)	1.4	(1.3-1.5)
**Civil status**					
married	4405	1.0		1.0	
cohabiting	5417	1.3	(1.2-1.3)	1.3	(1.2-1.4)
not cohabiting	578	2.8	(2.6-3.2)	2.5	(2.2-2.8)
**Education**					
primary	364	1.0		1.0	
secondary	3604	0.7	(0.6-0.8)	0.8	(0.7-0.9)
≥ 4 yr univer.	5494	0.5	(0.4-0.6)	0.6	(0.5-0.7)
> 4 yr univer.	938	0.8	(0.7-1.0)	0.9	(0.8-1.1)
**Parity**					
P0	5053	1.0		1.0	
P1+	5347	1.1	(1.1-1.2)	1.0	(1.0-1.1)
**BMI**					
<20	1245	1.0		1.0	
20-24.9	5642	1.0	(0.9-1.1)	1.0	(0.9-1.1)
25-29.9	2351	1.1	(1.0-1.1)	1.0	(0.9-1.1)
≤ 30	1162	1.2	(1.1-1.3)	1.0	(1.0-1.1)
**Smoking 1st trim.**					
no	8884	1.0		1.0	
sometimes	440	1.6	(1.5-1.8)	1.4	(1.2-1.6)
daily	1076	2.2	(2.0-2.4)	1.6	(1.5-1.8)
**Alcohol 1st trim.**					
never	8587	1.0		1.0	
<1 /week	1792	1.3	(1.2-1.3)	1.2	(1.1-1.2)
2-7 /week	21	3.2	(1.8-5.6)	2.5	(1.4-4.6)
**Child abuse**					
no	7183	1.0		1.0	
yes	3217	2.5	(2.4-2.6)	2.4	(2.3-2.5)

Crude and adjusted odds ratios (ORs) with 95% confidence intervals (CI), (N = 52,964).

*Numbers of exposed to adult abuse in each category. **30-34 is reference group in age category. Comparison group is no adult abuse. Analyzed for complete cases only.

## Discussion

Thirty two percent of the 65,393 pregnant women in our low-risk population reported any lifetime abuse. Adult and child abuse were reported by 20% and 19%, respectively, of whom around 30% reported exposure to two or three types of abuse. Living alone, exposure to child abuse, smoking and drinking alcohol in the first trimester, and being 35 years or older were associated with any adult abuse.

### Strengths and limitations

The large number of participants and the population-based design are major strengths of our study. Furthermore, women were subjected to a broad spectrum of questions and had no information that abuse reports would be linked to other questions. It is a strength of the study that the questions give information about exposure to three types of abuse (emotional, physical and sexual) in addition to information from a long time spectrum (childhood, adulthood and last 12 months exposure), as this gives a broad picture of the exposure to abuse in this population. Three abuse measures give more possible comparisons with other studies, as does the broad time aspect of the questions; as many studies only include one or two types of abuse, seldom three, and usually a shorter time aspect than in our study. A limitation to our study is that none of the four abuse questions in our study were validated when implemented, nor at the time of the start of the survey in 1999. There has been a huge development in the past decade in improving and acknowledging the importance of using validated instruments for research and screening in this field. Nevertheless, not many abuse instruments were validated prior to the start of the MoBa study. The questions on emotional abuse in the current study are similar to those in the NorVold questionnaire which was validated in 2002, but the populations in our study and the NorAQ study are not directly comparable, as the latter study includes patients from three gynecology clinics and one population based sample. The validation study showed that the abuse variables in the NorAQ have good reliability and validity [16]. This was the first validation study of an instrument in the Nordic countries, and the aim was to create an instrument making it possible to compare prevalence rates between the five Nordic countries [16]. Furthermore, there are great similarities between the question on sexual abuse and given response option in the current study, and the question of sexual abuse in the Abuse Assessment Screen (ASS) [21]. It does not have a well-established psychometric property, but it has a broad conceptualization of abuse. According to a review on abuse screening tools, no single tool had well established psychometric properties, including the ASS [21]. The question on physical abuse in our study gives room for subjective interpretation. Nevertheless, we decided to include the question because we wanted to show the broad aspect of reported abuse among our population. Anyhow, for each of the questions, whether the abuse is described well or not, the reported abuse is subjected to the woman’s interpretation of both the questions and her own experiences. As the information was available we thought it was better to use it rather than excluding it.

Our population is based on pregnant women from all over Norway. More than 90% of the women who agreed to participate in the Mother and Child Cohort Study (MoBa) responded to Questionnaires One and Three during pregnancy [14], indicating “dedicated” responders. In addition, only 493 women, (less than one percent) of the participating women, had not responded to any of the abuse questions in the questionnaire. This shows great willingness to respond about abuse exposure. Furthermore, of those who reported one or more types of abuse, almost all (98%) also reported on the identity of the perpetrator. A limitation of the study is the high rate of missing data for the questions on abuse in the preceding 12 months. A reason for this could be the way in which the questions were expressed (Figure 2). Most of the questions in the questionnaire required that the women indicated only if she had a positive answer to the specific question. On these particular questions on abuse during the last 12 months the women were required to change the way of responding by indicating yes or no. In addition these questions were at the very end of the questionnaire that had 94 main questions, with several sub questions.

Substantially more women reported emotionally abuse than other kinds of abuse in our study. It is probably easier to report emotional abuse than sexual and physical abuse. Another reason could be that our study contained two questions on emotional abuse compared with one question of sexual and physical abuse, respectively. The questions in our study allow women to define both “forced” and “sexual acts”, and “exposed to physical acts”. Some cases of sexual and physical abuse will not be identified by this question. The low overall response rate of 38.5% in the MoBa is a limitation. Nilsen et al. investigated this possible bias in the MoBa study by comparing women participating in the study with all women giving birth in Norway, and concluded that prevalence estimates of exposures and outcomes, but not estimates of exposure-outcome, were biased [15]. The same study showed that more women in the MoBa were living alone and fewer were under the age of 25 compared with all women giving birth in Norway. We would expect that these factors and the great number of highly educated women in the MoBa study contribute to a lower prevalence of abuse than in the general population. Retrospective reporting is a challenge, but difficult to avoid in these kinds of surveys. The women were on average 30 years old when responding to exposure to abuse. Their reporting on abuse could be subject to recall bias. Being pregnant could influence their response, as negative exposures denied earlier in life, could come to awareness. The way we see it this can both have a potential impact on depression, and oppositely, being in a depressed state may have an impact of memories and hence on the retrospective reporting.

### Comparing prevalence results to other studies

#### Lifetime exposure

In our study, 32% of subjects reported any lifetime abuse (emotional, physical and sexual). This is in the mid-range of the results in Devries et al’s study, where about 11% to 64% reported lifetime abuse. That study analyzed prevalence data of intimate partner violence from 19 countries, and reported higher prevalence in African and Latin American countries relative to European and Asian countries [22]. The only two developed countries in the study, Denmark and Australia, reported 22% and 27%, respectively, which is lower than our results. The data-collection method in the latter two countries was interviewing by telephone, while in the other countries, it was interviewing face-to-face. This may partly explain the differences within that study, as the first method is recognized as having lower response rates than face-to-face interviews, but not why the results differ from ours [22]. One possible reason might be that their study examined partner abuse, while in our study abuse from other perpetrators also is also reported. Reported lifetime abuse in the Gazmararian et al. review article of abuse during pregnancy varied from 10% to 30% [7], which is lower than any lifetime abuse reported in our study, but corresponds with lifetime physical abuse reported in our study at 11%. The study is from United States and other developed countries comparable with Norway, and focused mainly on physical abuse. Our results on lifetime physical abuse were lower than those reported in a Swedish study from three gynecology clinics and in one randomly selected population group, where women reported exposure to lifetime physical abuse in the range of 32% to 38% [23]. This may reflect the fact that clinical populations often report a higher prevalence than population-based studies [17,24]. One reason for this is that self-reported problems, both mental and physical, are associated with exposure to abuse [25]. A second reason is that health care utilization is higher among those exposed to abuse [26,27]. Third, high prevalence rates are seen in specific groups, for example, among women with severe menstrual syndrome [28] or pelvic pain [29]. Emotional abuse is reported more frequently than physical or sexual abuse [23,30-33], thereby contributing to a higher prevalence of any lifetime abuse in studies where questions about sexual, physical and emotional abuse are included. In addition, the current study also contained two questions about emotional abuse. This may have contributed additionally to the higher prevalence detected in our study compared with other studies on any lifetime abuse. The population-based design and extensive questionnaires in our study indicate a lower prevalence compared with studies focusing on abuse only, which are recognized as showing a higher prevalence than surveys designed with a broader perspective [4].

### Pregnancy related abuse

Our study gives information about exposure to abuse in the preceding 12 months, asked at about 30 weeks of gestation (Table 1). Hence, our study provides information about exposure to abuse prior to, or during pregnancy, and the results are regarded as pregnancy-related. Our findings on last-year prevalence of any abuse were 5%, corresponding with the first national Norwegian study in a non-obstetric population, where 6% reported any partner abuse in the preceding year [12]. Our results are, however, in the lower range of the findings in WHO’s multi-country study, where between 4% (Japan and Serbia and Montenegro) and 54% (Ethiopia) of the women reported exposure to partner abuse in the last 12 months [9]. Findings in this article showed that the prevalence of abuse is usually lower in industrialized settings than in rural settings [9]. Our results correspond with the lower prevalence rates reported in the latter study, and are also in the lower range of the findings from the Gazmararian et al’s review article on the prevalence of abuse of pregnant women in developed countries, which found that exposure to abuse in the preceding 12 months in four studies varied between 6% and 24% [7]. These differences in methodology may explain why our results correspond with the lower reported prevalences, in addition to the possibility that there is a real lower exposure to abuse in Norway as an industrialized country.

### Perpetrators

Our results showed that a known perpetrator is more frequent for all types of abuse (Figure 5). This finding corresponds well with other studies reported in pregnant populations [10]. WHO’s multi-country study suggests that women are at more risk of abuse from intimate partners than from any other [9]. The questionnaire in MoBa did not elicit information about a partner or former partner being the perpetrator, out of consideration for the women’s safety receiving and possibly filling out the questionnaire at home. Other research, however, suggests that this known person most frequently will have been the present or former partner [9].

### Background information and relation to abuse

Living alone, exposure to child abuse, drinking alcohol in the first trimester, and being 35 or older were associated with exposure to any adult abuse in our study. Women living alone were a small group in our sample, but interestingly, the study also showed a higher exposure to abuse in the cohabiting group compared with the married group (OR 1.3, 95% CI 1.2-1.4). Our results showing that living alone or being single was associated with a higher exposure to abuse and that being married or cohabiting was a protective factor correspond with another study [11]. Our results also agree with studies showing an association of exposure to child abuse [34] and of use of alcohol [34,35] with increased prevalence of reporting abuse, even though none of these studies can predict a causal connection between exposure to background factors and exposure to abuse. The cross–sectional design of our study provides associations and not causal relations. In the current study, women above 35 reported more exposure to any adult abuse than women in the other age groups. This may be due to accumulative effects, as the older subjects have had more time to be exposed to abuse. A Swedish clinical study showed the contrary, however, as high age was negatively associated with lifetime abuse in that study [23]. The WHO’s study on recent abuse reported higher exposure to abuse with lower age [34], and in Devries et al. study, prevalence of abuse during pregnancy was relatively constant to the age 35 and then slightly declined [22]. Younger age may reflect less opportunity to protect oneself and lower reporting from the eldest can be due to fading of memory with age. The literature is inconclusive regarding education and exposure to abuse. Norway has a generally high level of education and more women than men graduate at university level. In our study, we chose to divide higher education into two groups, those who completed four years of education at university level and those with more than four years. Our results showed that the association to any adult abuse was weaker in the group reporting four years of education at university level compared to all the other educational groups (Table 3). A low level of education is reported to be a risk factor for exposure to abuse in the populations-based WHO study on recent abuse [34], while a Swedish study from three clinical populations and one randomly selected population reported that educational level had a positive association with physical abuse but not with sexual abuse in both clinical and population samples [23]. One possible explanation is that women with higher education have higher self-esteem, are more aware of their rights, and tolerate less violation of their integrity [23]. Studies show that background factors have different impacts on different types of abuse. This indicates that the type of abuse (emotional, physical or sexual) and whether it is a single type or overlapping types are results of various patterns. Risk factors therefore vary depending on the type of abuse studied, as suggested by a study from Vietnam [30].

### Public health implications

Previous research has shown that abuse of women and children is associated with morbidity for the women and the children, possibly both with short and long term consequences. Studies, including the current, have reported that abuse of women is more frequent than many other pregnancy complications [10]. Five percent of the women in our study reported exposure to abuse in the last 12 months at Week 30 of gestation. This is comparable with the prevalence of preeclampsia (2-5%) and gestational diabetes (5%) in Norway, conditions for which pregnant women are routinely screened. Several studies have shown more negative reproductive health consequences in abused than in non-abused women, e.g. reporting more pregnancy terminations [11], and more pregnancy complaints and fear of birth [20,36]. Self-reported poor health and psycho-somatic symptoms are also more common in abused than non-abused women [19]; so also with symptoms of chronic pelvic pain, stomach pain, headache, emotional distress and depression [12,25,37,38].

## Conclusions

Our study provides information from pregnant women about self-reported exposure to adult and child abuse within a population with relatively few risk factors for abuse. Whether screening for abuse should be incorporated into routine antenatal care is an important discussion, but is beyond the limits of this article. Antenatal care is free in Norway and almost all women participate in regular check-ups at their general practitioner and/or midwife. Pregnancy may be the only time when healthy women come into frequent regular contact with health care providers, creating a good opportunity to ask about the experience of abuse and to identify those at risk.

## Competing interests

There are no potential conflicts of interests. There are no financial competing interests. No one have in the past five years received reimbursements, fees, funding, or salary from an organization that may in any way gain or lose financially from the publication of this manuscript, either now or in the future.

## Authors’ contributions

MFS prepared the data, performed the statistical analyses, and drafted and corrected the manuscript. HG contributed on the interpretation of the analyses and helped to draft and critically revised the manuscript. JHB advised on the statistical analyses and the interpretation of results and drafted the manuscript. BS conceived the study idea and planned the study and contributed to the interpretation of the analyses and the drafting of the manuscript. ML contributed to the preparation of the data and the interpretation of the results and drafted and critically revised the manuscript. All authors contributed to the study’s design and read and approved the final manuscript.

## Pre-publication history

The pre-publication history for this paper can be accessed here:

http://www.biomedcentral.com/1471-2458/13/186/prepub
